# Beyond Weight Loss: Early Real-World Evidence of Semaglutide in Obesity

**DOI:** 10.3390/medicines13030021

**Published:** 2026-06-28

**Authors:** Steluța Constanța Boroghină, Amalia-Ioana Arhire, Teodora Papuc, Miruna Sînziana Chiper, Diana-Andreea Meluță, Sorana Maria Pîrcălabu, Roxana Andreea Dănăilă, Mădălina Cristache, Carmen Gabriela Barbu

**Affiliations:** 1 Kilostop Junior Nutrition Clinic, 010073 Bucharest, Romania; steluta.boroghina@umfcd.ro (S.C.B.); miruna.chiper@kilostopjunior.ro (M.S.C.); diana.meluta@kilostopjunior.ro (D.-A.M.); sorana-maria.pircalabu@rez.umfcd.ro (S.M.P.); roxana-andreea.danaila@mst.umfcd.ro (R.A.D.); madalina.cristache@kilostopjunior.ro (M.C.); 2 General Medicine Faculty, “Carol Davila” University of Medicine and Pharmacy, 050474 Bucharest, Romania; carmen.barbu@umfcd.ro; 3 Department of Pediatrics, Fundeni Clinical Institute, 022318 Bucharest, Romania; 4 Endocrinology and Diabetes, Elias Emergency University Hospital, 011461 Bucharest, Romania; 5 Department of Pediatrics, Marie Curie Hospital, 077120 Bucharest, Romania

**Keywords:** pediatric obesity, obesity treatment, GLP-1 RAs, metabolic syndrome, real-world evidence

## Abstract

**Background:** Obesity is a chronic, relapsing disease that often proves resistant to lifestyle measures alone. Glucagon-like peptide-1 receptor agonists (GLP-1 RAs), are reshaping treatment, yet prospective real-world data remain limited. **Objective:** To prospectively assess the effects of once-weekly Semaglutide on weight, body composition, and metabolic health in obesity. Methods: An exploratory observational study of 37 patients initiating Semaglutide (mean age 31 years; 11 children and adolescents; 22 females) was conducted. All met obesity criteria (baseline BMI 34.7 kg/m^2^). Anthropometry, bioimpedance body composition, and fasting biochemistry were obtained at baseline and 3 months. Variables were reported as mean ± SD or median (IQR) according to normal/non-normal distribution, whether a parametric test or a Wilcoxon one was used. Parametric or non-parametric paired tests (two-sided α = 0.05) were applied. We also explored tri-ponderal mass index (TMI, kg/m^3^) and its correlations with metabolic markers. **Results**: At 3 months, body weight decreased by a median 8.0 kg (*p* < 0.001), BMI by 1.6 kg/m^2^ (*p* < 0.001). Body fat percentage declined: 43.4% to 42.8% (*p* = 0.009), with a small reduction in skeletal muscle mass (−0.6 kg; *p* = 0.035). Fasting glucose improved (*p* = 0.030) and HOMA-IR fell significantly. HbA1c changes were minimal, consistent with near-normal baseline values. Triglycerides decreased, while total cholesterol, LDL-C, HDL-C, liver enzymes, creatinine, uric acid, and 25-OH vitamin D remained stable. Baseline TMI (median 20.13 kg/m^3^; IQR 3.80) correlated strongly with HOMA-IR (r = 0.766, *p* < 0.001) and moderately-to-strongly with body fat percentage (r = 0.621, *p* < 0.001). **Conclusions:** In this real-world cohort, Semaglutide produced rapid, clinically meaningful improvements in weight, adiposity, and insulin resistance within 3 months. Findings suggest that Semaglutide may represent a promising adjunct to lifestyle therapy in obesity management.

## 1. Introduction

### 1.1. The Pediatric Obesity Challenge

Pediatric obesity is a chronic, progressive, and relapsing disease that clusters with dysglycemia, dyslipidemia, hypertension, and fatty liver disease, and adversely affects mental health and quality of life. Although lifestyle modification remains the bedrock of care, the durability of weight loss achieved through diet, physical activity, and behavioral counseling alone is limited for many patients, underlining the need for effective adjuncts within a long-term, chronic-care framework [1,2]. In recent years, a convergent body of evidence has emphasized that pharmacotherapy can support sustained weight loss and cardiometabolic benefit when layered onto multidisciplinary care [3].

The global burden of obesity continues to increase globally. Recent international estimates indicate that more than 1 billion people worldwide are living with obesity, including over 160 million children and adolescents, with prevalence rates continuing to increase across both high-income and middle-income countries. Current projections indicate that overweight and obesity may affect nearly 20% of children worldwide by 2030 if existing epidemiological trends persist. Pediatric obesity is associated not only with adverse metabolic and psychological outcomes, but also with increased healthcare utilization and long-term economic impact. Recent analyses from JAMA Pediatrics (2025) identified higher lifetime healthcare expenditures among individuals with pediatric obesity, primarily related to cardiometabolic complications and associated comorbid conditions [4]. These findings underscore that childhood obesity is not merely a transient developmental condition but a chronic disease with long-term economic and social consequences.

Although randomized controlled trials (RCTs) provide high-level evidence regarding efficacy and safety under controlled conditions, their strict inclusion criteria, standardized lifestyle interventions, and intensive monitoring protocols may limit generalizability to routine clinical practice. Real-world data are therefore essential to understand treatment effectiveness in heterogeneous populations, including patients with varying socioeconomic backgrounds, adherence patterns, comorbidities, and access to multidisciplinary care. In pediatric obesity in particular, real-world evidence helps clarify tolerability, dose escalation feasibility, and metabolic responses outside the rigid structure of clinical trials. Such data are critical for informing everyday decision-making in specialized obesity clinics and for guiding policy-level implementation of pharmacotherapy in broader healthcare systems [5].

### 1.2. Real-World Evidence and Rationale for a Mixed-Age Cohort

Data from Central and Eastern Europe regarding pharmacological obesity management remain limited, despite increasing obesity prevalence and growing use of GLP-1 receptor agonists in clinical practice. According to the WHO European Regional Obesity Report, substantial disparities persist across the region regarding access to multidisciplinary obesity care and evidence-based anti-obesity pharmacotherapy [6]. Consequently, prospective real-world studies reflecting routine clinical practice are needed to complement randomized controlled trial evidence.

In specialized obesity clinics, adolescents transitioning into adulthood are frequently managed within integrated multidisciplinary services together with young adults [7]. Although physiological differences exist between pediatric and adult populations, both groups share common obesity-related metabolic disturbances, including excess adiposity, insulin resistance, dyslipidemia, and increased cardiometabolic risk. Because the present study aimed to evaluate early treatment-associated metabolic and anthropometric changes under routine clinical conditions, inclusion of both adolescent and adult participants was considered appropriate. To address potential heterogeneity, subgroup analyses according to age category were additionally performed.

### 1.3. GLP-1 Receptor Agonists in Obesity Management

GLP-1 receptor agonists have emerged as effective adjunctive therapies for obesity management by promoting weight reduction and improving metabolic parameters across different age groups. In adolescents with obesity, once-weekly Semaglutide combined with lifestyle intervention has demonstrated significantly greater BMI reduction compared with lifestyle intervention alone [8]. Similarly, adult randomized trials and emerging real-world studies have reported clinically relevant reductions in body weight, adiposity, and insulin resistance during Semaglutide treatment [9].

GLP-1 receptor agonists promote weight reduction through multiple complementary mechanisms, including appetite suppression, delayed gastric emptying, reduced caloric intake, and modulation of central satiety pathways [10,11,12,13]. In addition, these agents improve glucose homeostasis by enhancing glucose-dependent insulin secretion and suppressing glucagon release. Collectively, these physiological effects contribute to reductions in adiposity and improvement of obesity-related metabolic dysfunction.

### 1.4. Current Evidence in Children and Adolescents

Multiple systematic reviews and meta-analyses in youth consistently demonstrate that GLP-1 RAs produce superior reductions in body weight and BMI versus lifestyle or placebo, with generally acceptable safety profiles dominated by transient gastrointestinal symptoms [1,10,11,12]. Collectively, randomized and real-world data support GLP-1 RAs as effective adjuncts to multidisciplinary care for pediatric obesity, provided dosing, monitoring, and education are embedded within a structured program [8,9,14,15].

### 1.5. TMI and Body Composition Assessment

Assessment of adiposity during growth and weight reduction remains challenging, particularly in adolescents, because BMI alone does not fully reflect body composition changes. The tri-ponderal mass index (TMI; kg/m^3^) has been proposed as a complementary anthropometric marker that may better correlate with adiposity and metabolic risk during adolescence compared with BMI alone [16,17]. Therefore, exploratory analyses of TMI were included in the present study to further characterize relationships between adiposity and insulin resistance during Semaglutide treatment.

### 1.6. Study Hypothesis and Objectives

We hypothesized that once-weekly Semaglutide administered under routine multidisciplinary obesity care would be associated with early improvements in body weight, adiposity, and metabolic parameters after 3 months of treatment.

Accordingly, we conducted an exploratory prospective observational study in a mixed-age clinical cohort to evaluate early changes in body weight, body composition, and metabolic markers following Semaglutide initiation. The primary endpoint was change in body weight at 3 months. Secondary endpoints included changes in BMI, body fat percentage, insulin resistance markers, lipid profile, and selected biochemical safety parameters. Exploratory analyses additionally evaluated associations between TMI, adiposity, and insulin resistance within the study population.

## 2. Materials and Methods

### 2.1. Study Design

This was an exploratory prospective observational study conducted in the nutrition clinic Kilostop Junior Bucharest, Romania, a specialized center for pediatric and adult obesity management and the Elias Hospital, Bucharest, Romania. The study included patients consecutively enrolled between May and September 2025.

### 2.2. Outcomes

The primary outcome of the study was the change in body weight from baseline to 3 months. Secondary outcomes included changes in BMI, body composition parameters (percent body fat and skeletal muscle mass), glycemic markers (fasting glucose, insulin levels, HOMA-IR, HbA1c), lipid profile, and exploratory correlations between TMI and metabolic parameters.

No formal sample size calculation was performed due to the exploratory nature of the study. The sample size was determined by the number of consecutive eligible patients initiating Semaglutide during the study period.

### 2.3. Study Population

Eligible participants were individuals diagnosed with obesity, without known diabetes, who initiated pharmacological treatment with Semaglutide. Inclusion criteria were: diagnosis of obesity according to age and sex adjusted BMI criteria, availability of complete baseline anthropometric and biochemical measurements and at least one follow-up visit at 3 months. Patients with diabetes or missing essential data were excluded. All eligible patients presenting to the participating centers during the study period were consecutively included in the study. A total of 37 patients met the inclusion criteria: 11 children and adolescents (<18 years) and 26 adults, from whom 22 females and 15 males with varying degrees of obesity at baseline.

The study adhered to the Helsinki Declaration of 2013, with parental approval obtained through written informed consent for the use of medical records in scientific research.

### 2.4. Intervention

All participants received Semaglutide treatment subcutaneously every week, titrated according to international obesity treatment guidelines and the manufacturer’s Summary of Product Characteristics. Dose escalation followed a standardized 16-week schedule: 0.25 mg once weekly during weeks 1–4, increased to 0.5 mg once weekly during weeks 5–8, then 1.0 mg once weekly during weeks 9–12, to 1.7 mg once weekly during weeks 13–16, and finally to the maintenance dose of 2.4 mg once weekly from week 17 onwards. Each step was scheduled at 4-week intervals to allow gastrointestinal adaptation. If a patient experienced clinically significant adverse effects (most commonly persistent nausea, vomiting or diarrhea) at a given step, the up-titration was postponed for an additional 4 weeks at the same dose, or the dose was temporarily reduced to the previous level until symptoms resolved. Patients who could not tolerate further escalation were maintained on the highest tolerated dose, in accordance with the Summary of Product Characteristics. The mean achieved dose at the 3-month visit and individual adherence to the titration schedule are reported in the Results section.

In addition, all patients were advised to follow a hypocaloric diet and structured physical activity recommendations, consistently with lifestyle interventions used in major Semaglutide obesity trials [9,14] which included approximately 150 min/week of moderate-intensity physical exercise.

### 2.5. Measurements

- Anthropometry and Body Composition

Body weight, BMI, body fat percentage (PBF), and skeletal muscle mass (SMM) were measured using a validated InBody 270 bioelectrical impedance analyzer, while resting metabolic rate (RMR) was measured by indirect calorimetry with an indirect calorimeter Fitmate manufactured by COSMED, Rome, Italy [18].

In addition to standard anthropometric indices, the tri-ponderal mass index (TMI) was calculated for each participant as weight (kg) divided by height (m^3^).

Measurements were obtained at baseline (0 months), after 3 months, and in a subset of patients also at 6 months.

- Biochemical assessments

A 10 mL peripheral blood sample collection after an overnight fast of at least 8 h, was made for biochemical analyses. The blood chemistry analysis included a glycemic profile, lipid, hepatic, renal profiles and 25 OH vitamin D.

The glycemic panel included fasting plasma glucose (enzymatic colorimetry, Architect c8000; Abbott Diagnostics, Abbott Park, IL, USA, kit 67921UQ02), glycated hemoglobin—HbA1c (HPLC on Bio-Rad D-10, Hercules, CA, USA with internal calibrator/controls), and fasting insulin (ECLIA on a validated platform). Insulin resistance and β-cell function were derived as HOMA-IR and HOMA-B from fasting insulin (μU/mL) and glucose (mmol/L) according to Matthews et al. [19].

The lipid profile—total cholesterol (kit 72291UD00), HDL-C (67136UQ10), triglycerides (71068UD00) and calculated LDL-C—was measured by enzymatic colorimetry on the Architect c8000 with manufacturer-recommended quality controls.

Hepatic enzymes (AST, kit 70068UD00; ALT, 72494UD00) were assessed by enzymatic colorimetry on the same platform.

Serum 25-hydroxy vitamin D was quantified by ELISA on the Chemwell 2010 (Awareness Technology Inc., Palm City, FL, USA) using the Immunodiagnostic Systems kit (AC-57SF1). The renal/mineral panel comprised serum creatinine (kit 71560UD00) and total calcium (74372UD00) on the Architect c8000, and inorganic phosphorus on the Vitros 5,1 FS Chemistry Analyzer (Ortho Clinical Diagnostics, Raritan, NJ, USA; kit 1203-0398-1506).

All assays followed manufacturers’ instructions and internal quality-assurance procedures.

### 2.6. Clinical Protocol, Baseline Assessment and Follow-Up Procedures

At study entry (S0), all participants underwent a standardized baseline evaluation performed within routine clinical care in a specialized obesity management setting. The initial assessment included a detailed medical and weight history, focusing on age of obesity onset, weight trajectory over time, previous lifestyle or pharmacological weight-loss interventions, and individual motivation for weight reduction. A comprehensive anamnesis was obtained for all participants, including personal pathological history, known allergies, current and prior medication use, and family history of obesity, type 2 diabetes, cardiovascular disease, or other relevant metabolic conditions. In pediatric and adolescent participants, additional information was collected regarding birth history, early growth patterns, developmental milestones, and pubertal development.

Baseline clinical evaluation primarily focused on obesity-related parameters. Anthropometric measurements were obtained using standardized procedures and included body weight, height, BMI, waist circumference, and body composition analysis. In pediatric and adolescent participants, growth and developmental status were additionally evaluated as part of routine clinical assessment. Height and weight trajectories were reviewed using age- and sex-appropriate growth charts, and pubertal development was assessed by the attending clinician according to Tanner staging criteria during routine physical examination. Tanner staging was used to contextualize body composition and metabolic findings relative to pubertal maturation, given the known physiological changes in adiposity, insulin sensitivity, and growth occurring during adolescence.

Clinical evaluation also included assessment of obesity-related comorbidities, previous weight-management interventions, current medication use, and relevant family history. Prior to initiation of pharmacological treatment, all participants were screened for contraindications to GLP-1 receptor agonist therapy, including personal or family history suggestive of medullary thyroid carcinoma or multiple endocrine neoplasia type 2, history of pancreatitis, severe gastrointestinal disease, or other conditions in which GLP-1 receptor agonists are not recommended.

A comprehensive nutritional assessment was conducted at baseline for all participants. Dietary evaluation included detailed characterization of habitual food intake, encompassing meal timing, food choices, portion sizes, frequency of meals and snacks, eating environment, and eating behaviors such as grazing or emotional eating. Participants were asked to complete a structured food diary, which was reviewed during follow-up visits. Physical activity habits were documented, including the type, frequency, and duration of structured exercise and daily physical activity.

Individual energy requirements were calculated for each participant based on the RMR measured by indirect calorimetry, and adjusted according to reported physical activity levels. Dietary recommendations were individualized, aiming to induce a clinically appropriate caloric deficit, generally corresponding to an approximate reduction of 500 kcal/day where feasible, or adjusted according to measured metabolic rate, age, and clinical context. In pediatric participants, dietary prescriptions accounted for growth and developmental needs. Macronutrient distribution was prescribed within approximate target ranges (generally 45–50% carbohydrates, 20–25% protein, and the remainder from fats), with individual adaptations based on metabolic profile, comorbidities, and nutritional needs. Participants were instructed to maintain ongoing food and physical activity logs throughout the follow-up period. Individuals identified as requiring additional psychological or behavioral support were referred to a psychologist as part of multidisciplinary care.

Semaglutide was administered subcutaneously once weekly and titrated according to internationally accepted obesity treatment protocols, with age-appropriate dosing schedules for adults and pediatric patients. Dose escalation followed the 16-week schedule detailed above (0.25, 0.5,1.0, 1.7, 2.4 mg, with 4-week steps), with individual adjustments of the up-titration pace based on tolerability. In participants younger than 18 years, treatment initiation and follow-up were conducted with the involvement of a parent or legal guardian, who attended visits and provided consent for treatment and data use.

Follow-up visits followed a structured schedule designed both to support adherence to nutritional and physical activity recommendations and to ensure safe pharmacological titration. During the first 4 weeks of treatment, patients were contacted weekly, either by remote consultation or by a face-to-face visit at the clinic, to evaluate tolerability of the starting dose, reinforce lifestyle recommendations, and confirm correct injection technique. From weeks 5 to 16, corresponding to the dose escalation phase, patients were reviewed every 2 weeks, with anthropometric measurements and a structured tolerability assessment performed before each dose increase. Once the maintenance dose, or the highest individually tolerated dose was attained, visits were spaced to monthly intervals, and included full anthropometric, body composition and clinical reassessment, together with a review of dietary and physical activity adherence. Between visits, participants were encouraged to report any symptoms or concerns through a dedicated clinic contact channel.

Biochemical assessments were repeated at 3 months and, in a subset of participants, at 6 months, using the same standardized laboratory methods as at baseline. Although patients were free to report adverse events spontaneously at any clinical encounter, safety surveillance did not rely on spontaneous reporting alone: it was conducted prospectively, through a structured, multimodal approach. At every follow-up visit, the attending clinician administered a standardized adverse event checklist that systematically queried the most frequent GLP-1 receptor agonist-related symptoms, including nausea, vomiting, diarrhea, constipation, abdominal pain, dyspepsia, eructation, early satiety, fatigue, headache, dizziness, palpitation and injection site reactions. Additional directed questions screened for clinical features suggestive of acute pancreatitis (persistent severe epigastric pain radiating to the back, with or without vomiting), biliary disease (right upper quadrant pain, jaundice, post-prandial pain) and dehydration. Each event was graded using CTCAE v5.0, alongside its temporal relationship to dose escalation and the resulting action (continue, postpone, reduce or discontinue). Laboratory safety was built into the 3-month and 6-month biochemistry panels, which covered serum lipase and amylase, liver enzymes (AST, ALT, GGT) and total bilirubin, creatinine, and a complete blood count. Predefined thresholds on any of these tests prompted an abdominal ultrasound whenever pancreatitis or cholelithiasis was suspected. Blood pressure and heart rate were checked at every in-person visit. No treatment interruptions or permanent discontinuations occurred during the observation period.

This structured clinical protocol reflects real-world multidisciplinary obesity care and was designed to ensure individualized treatment, safe pharmacological titration, and systematic monitoring of anthropometric and metabolic outcomes during Semaglutide therapy.

### 2.7. Statistical Analysis

Given the observational design, small sample size, and absence of a control group, this study should be considered exploratory in nature.

Statistical analyses were performed using IBM SPSS Statistics for Windows, Version 26.0, IBM Corp., Armonk, NY, USA. Only participants with both baseline and 3-month data available were included in the paired analyses. Normality of paired differences (0–3 months) was assessed using the Shapiro–Wilk test. Variables with normal distribution were analyzed using paired *t*-tests and presented as mean ± SD. Non-normal variables were analyzed using Wilcoxon signed-rank tests and presented as median [IQR]. Subgroup analyses compared: children and adolescents versus adults, male versus female, using independent samples *t*-tests or Mann–Whitney U tests, according to normality. A two-sided significance level of *p* < 0.05 was considered statistically significant.

Because no international WHO/CDC reference percentiles exist for TMI, descriptive analyses were performed relative to the study cohort. Scatter-plots with linear regression were generated to explore associations between TMI and clinically relevant metabolic variables, including HOMA-IR, body fat percentage, and resting metabolic rate (RMR). Pearson correlation coefficients and *p*-values were computed.

## 3. Results

Thirty-seven individuals with obesity were enrolled (22 females, 15 males; mean age 31 years, range 11–60), including 11 participants < 18 years. The mean achieved Semaglutide dose at 3 months was 1 mg/week. Adherence to treatment was assessed based on follow-up attendance and patient self-report, with no treatment discontinuations recorded.

Glycemic indices reflected insulin resistance (fasting glucose ~93–95 mg/dL, fasting insulin generally >15 μU/mL, median HOMA-IR 3.8–5.0), while lipid profile, liver enzymes, uric acid, creatinine, and 25-OH vitamin D fell within ranges expected for an obesity clinic population.

At baseline: Median body weight was 90.5 kg [77.7–103.3], while mean BMI was 34.7 ± 6.3 kg/m^2^ (95% CI: 32.6–36.8). Body fat percentage (PBF) averaged 43.4% (95% CI: 41.6–45.2), and skeletal muscle mass (SMM) was 29.6 kg [29.35–29.85]. Resting metabolic rate was 1276 kcal/day [980.8–1571.2].

At 3 months: BMI decreased from 34.7 ± 6.3 kg/m^2^ (95% CI: 32.6–36.8) to 33.1 ± 6.2 kg/m^2^ (95% CI: 31.1–35.1), corresponding to a mean reduction of −1.6 kg/m^2^ (95% CI: −2.1 to −1.1, *p* < 0.001). PBF decreased from 43.4% (95% CI: 41.6–45.2) to 42.8% (95% CI: 41.0–44.6) (*p* = 0.009). Fasting glucose decreased from approximately 94 mg/dL to 89 mg/dL (mean difference −5 mg/dL, 95% CI: −9 to −1, *p* = 0.030).

In the subgroup analyses: The mean BMI reduction was −0.76 kg/m^2^ (95% CI: −2.1 to 0.6) in youth vs. −1.47 kg/m^2^ (95% CI: −2.5 to −0.4) in adults (*p* = 0.23). The mean PBF reduction was −0.13% (95% CI: −0.8 to 0.5) vs. −1.19% (95% CI: −2.0 to −0.4) (*p* = 0.16).

**Figure 1:**
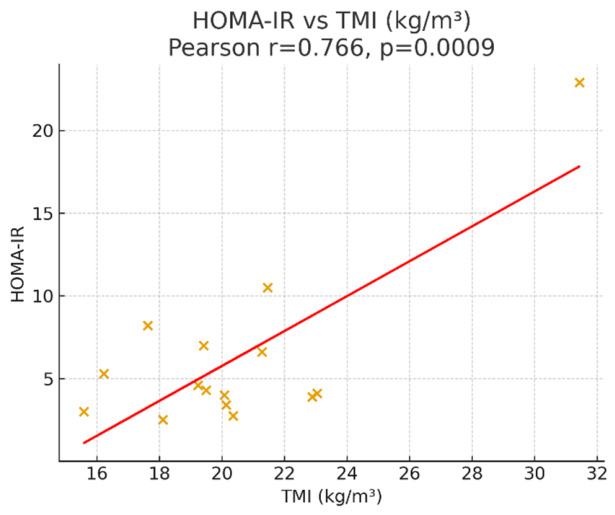
Strong correlation between HOMA-IR and TMI. Each orange marker (×) represents an individual patient; the red line denotes the linear regression (line of best fit) describing the relationship between TMI and HOMA-IR.

**Figure 2:**
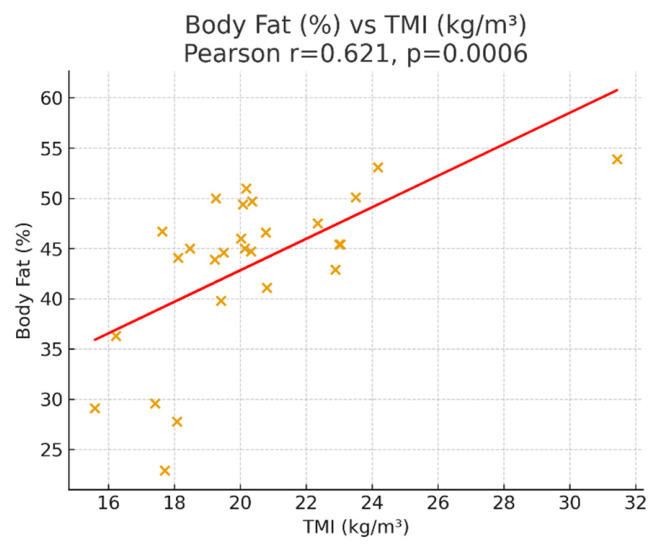
Moderate correlation between PBF and TMI. Each orange marker (×) represents an individual patient; the red line denotes the linear regression (line of best fit) describing the relationship between TMI and PBF.

At baseline, the TMI was 20.13 kg/m^3^ (median; IQR 3.80; internal P5–P95 17.17–28.16 kg/m^3^), indicating wide adiposity variability; TMI correlated strongly with HOMA-IR (r = 0.766, *p* < 0.001) as seen in Figure 1 and moderately-to-strongly with PBF (r = 0.621, *p* < 0.001) as seen in Figure 2 and showed a negative, non-significant association with RMR (r = −0.686; *p* = 0.519).

In the pediatric subgroup (*n* = 11), baseline BMI was high (median 33.95 kg/m^2^; IQR 4.90), placing all participants in the overweight/obesity range by CDC BMI-for-age references, with most meeting criteria for obesity.

After 3 months of once-weekly Semaglutide with lifestyle counseling, body weight decreased significantly (median −8.0 kg; Wilcoxon *p* < 0.001) and BMI fell from 34.7 ± 6.3 to 33.1 ± 6.2 kg/m^2^ (mean Δ ≈ −1.6 kg/m^2^; paired *t*-test *p* < 0.001), SMM decreased slightly from 29.6 to 29.0 kg (Wilcoxon *p* = 0.035) and HOMA-IR decreasing to 3.1–4.2 (Wilcoxon *p* < 0.05) as seen in Table 1.

Body composition improved modestly: PBF declined from 43.4% to 42.8% (*p* = 0.009) and RMR showed a small, non-significant downward drift consistent with metabolic adaptation. Glycemic control improved, with fasting glucose falling to ~88–90 mg/dL (*p* = 0.030), fasting insulin trending downward, and HOMA-IR decreasing to 3.1–4.2 (Wilcoxon *p* < 0.05), while HbA1c changed little (5.5–5.7% to 5.4–5.5%), in line with near-normal baseline values and exclusion of diabetes as seen in Table 2.

**Figure 3:**
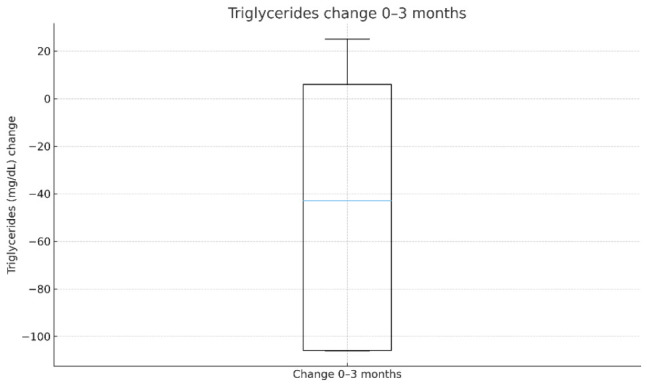
Improvement of triglycerides at 3 months of obesity treatment.

Triglycerides fell significantly (*p* < 0.05 by paired testing as appropriate), as seen in Figure 3, whereas total cholesterol, LDL-C, and HDL-C were unchanged, yielding an overall stable lipid profile. No significant shifts were observed in AST, ALT, GGT, creatinine, uric acid, or 25-OH vitamin D.

Subgroup analyses suggested broadly similar early responses across age and sex: in youth versus adults, numerical differences in weight (−2.38 vs. −3.79 kg; *p* = 0.40), BMI (−0.76 vs. −1.47 kg/m^2^; *p* = 0.23), and PBF (−0.13% vs. −1.19%; *p* = 0.16) were not statistically significant, and no sex-based differences emerged for anthropometric or metabolic outcomes.

In a small subset with 6-month data (*n* = 7), a continued improvement in weight and metabolic measures was observed, although the sample was too small for robust inference.

## 4. Discussion

In this exploratory observational, real-world cohort including adolescents and adults with obesity, once-weekly Semaglutide produced rapid reductions in body weight and BMI over three months, accompanied by early improvements in insulin resistance and a modest decline in percent body fat. Skeletal muscle mass decreased slightly and routine biochemical indices—including liver enzymes, renal markers, uric acid, lipids (except triglycerides) and 25-OH vitamin D—remained broadly stable. Taken together, these observations align with the evolving evidence that GLP-1 RAs deliver clinically meaningful weight loss and metabolic benefits beyond lifestyle therapy alone.

Our magnitude of change at 3 months is directionally consistent with—but understandably smaller than—results from the STEP TEENS randomized trial, where adolescents receiving once-weekly Semaglutide 2.4 mg plus lifestyle intervention achieved substantially greater BMI and weight reductions vs. lifestyle alone and saw broader cardiometabolic gains (waist circumference, HbA1c, lipids, ALT), albeit with more gastrointestinal events (GI) and some cholelithiasis [14]. Our shorter follow-up and lower typical doses in routine care plausibly explain the smaller absolute changes, and they also reflect how Semaglutide is introduced and titrated in many services.

Real-world pediatric data now complement trial evidence. A UK tertiary-care observational series in 10–18-year-olds treated with once-weekly Semaglutide (final dose 1 mg) showed significant declines in BMI SDS and body weight at 6 and 12 months, with mostly mild GI adverse effects and a single case of gallstones [20]. Similar signals are emerging at population scale: A nationwide analysis from Israel reported that anti-obesity medications (AOMs)—including GLP-1 RAs—were associated with reductions in BMI z-scores and improvements in glycaemia and lipids during treatment in 10–18-year-olds, though benefits partially regressed after cessation [21]. These data frame our findings within a broader pattern: GLP-1 RAs are effective adjuncts in youth when layered onto lifestyle care, while durability after discontinuation remains a challenge.

Semaglutide engages central GLP-1 receptors to enhance satiety and diminish hunger and peripherally slows gastric emptying—changes that favor negative energy balance and preferential mobilization of visceral fat; in insulin-resistant states, GLP-1 RAs also augment insulin secretion and suppress glucagon, improving post-prandial glycaemia [20]. Our observed early fall in fasting glucose and HOMA-IR is therefore biologically coherent. The modest decline in resting metabolic rate and the small reduction in skeletal muscle mass likely reflect expected adaptive thermogenesis and loss of fat-free mass with weight reduction; importantly, we did not detect adverse shifts in liver or kidney function over three months.

Safety in our cohort was acceptable and is consistent with prior studies. In STEP TEENS, GI adverse events were more frequent with Semaglutide than placebo, but permanent discontinuations due to GI disorders were low, and growth and pubertal development appeared unaffected during the 68-week treatment period [14]. In the UK real-world series, GI symptoms were common yet generally mild, with rare cholelithiasis [20]. Our routine biochemistry also remained stable, which is reassuring for early safety in a mixed-age clinic setting.

Beyond weight, we explored TMI as a descriptive marker. We found strong positive correlations between TMI and HOMA-IR and moderate-to-strong correlations with PBF, suggesting TMI captured clinically relevant adiposity and metabolic risk within our sample [16,17]. This is salient because pediatric and adolescent body-composition assessment is imperfect when relying on BMI alone; as highlighted in pediatric services, BMI has recognized limitations for describing body composition, and complementary metrics (bioimpedance, DXA, or indices like TMI) can add context. Our data adds pragmatic support for incorporating TMI into routine characterization, especially during early pharmacotherapy when rapid changes may differentially affect fat and lean compartments.

A key translational question is durability. In adolescents, STEP TEENS did not include a long off-treatment follow-up, but adult STEP trials show that Semaglutide’s effect can persist with ongoing therapy and tends to regress after withdrawal—mirroring patterns seen with other AOMs [14]. Our 6-month subset hinted at continued improvement, but the sample was small; longer, structured follow-up with attention to dose optimization, adherence, and maintenance strategies (nutrition, activity, behavioral support) is warranted. As the UK series notes, some non-responders at 1 mg might benefit from higher obesity-specific doses (e.g., 2.4 mg), pending availability and regulatory pathways for youth [20].

This study has practical implications for mixed pediatric–adult clinics. First, the early metabolic gains (triglyceride reduction; HOMA-IR improvement) suggest that initiating Semaglutide may rapidly modify cardiometabolic risk even before large absolute weight losses occurs, consistent with trial observations where cardiometabolic risk factors improved alongside weight loss [14]. Second, our absence of signal in liver and renal biochemistry over three months aligns with pediatric trial safety and with real-world pediatric experience, though clinicians should remain vigilant for GI intolerance and gallstone risk. Third, our age- and sex-stratified analyses did not detect meaningful differences at three months, reinforcing that early treatment effects may be broadly similar across demographic groups in routine practice—an observation coherent with trial populations, albeit recognizing that STEP TEENS’ generalizability is constrained by sample composition [14,20].

A distinctive aspect of this study was the inclusion of both adolescent and adult participants within a single analytical framework. Although randomized clinical trials typically apply strict age stratification, real-world obesity care frequently operates along a continuum model, particularly during the transition from pediatric to adult services. This pragmatic design allowed assessment of early treatment-associated responses across developmental stages under comparable clinical conditions. Nevertheless, cohort heterogeneity may also have introduced biological and behavioral variability related to pubertal status, hormonal milieu, body composition dynamics, and lifestyle adherence. Consequently, subgroup findings should be interpreted cautiously and considered hypothesis-generating rather than definitive evidence of equivalence between age groups.

This study has several limitations that should be considered when interpreting the findings. First, the cohort was heterogeneous, including both adolescents and adults, with variability in baseline anthropometric characteristics, metabolic profiles, pubertal status, and developmental stages. Such heterogeneity reflects real-world clinical practice but may introduce additional variability in treatment response and limit the ability to detect subtle differences between subgroups.

The exploratory observational design, absence of a control group, and combined lifestyle intervention limit causal interpretation of the findings. In addition, variability in achieved Semaglutide dose and adherence may have influenced outcomes. Although safety monitoring was prospective and systematic, the study was not conducted within a randomized controlled framework. Finally, body composition was assessed using bioelectrical impedance analysis rather than imaging-based methods such as DXA.

In summary, our real-world findings show that Semaglutide initiates clinically meaningful early improvements in weight, adiposity, and insulin resistance within three months without adverse shifts in routine laboratory markers. Coupled with randomized adolescent data and emerging pediatric real-world evidence [21], these results support Semaglutide as a promising adjunctive therapy within multidisciplinary obesity care for obesity in youth and adults, while underscoring the importance of dose-appropriate titration, careful safety monitoring and long-term maintenance strategies to sustain benefits after the initial response, while highlighting the need for larger controlled studies and longer follow-up.

## Figures and Tables

**Table 1:** Changes in anthropometric and metabolic parameters.

Parameter	Baseline	3 Months	Change	*p*-Value
Weight (kg)	90.5 [77.7–103.3]	82.5 [69.7–95.4]	−8.0	<0.001
Skeletal Muscle Mass (SMM) (kg)	29.6 [29.35–29.85]	29.0 [28.75–29.25]	−0.6	0.035
HOMA-IR	4.4 [3.8–5.0]	3.7 [3.1–4.2]	−0.7	<0.05

Data are presented as median [IQR]. Wilcoxon signed-rank tests were used for all variables in this table. Full cohort (*n* = 37); youth subgroup (*n* = 11), adults (*n* = 26).

**Table 2:** Changes in anthropometric and metabolic parameters (mean ± SD or mean with 95% CI).

Parameter	Baseline	3 Months	Mean Difference (95% CI)	*p*-Value
BMI (kg/m^2^)	34.7 ± 6.3 (32.6–36.8)	33.1 ± 6.2 (31.1–35.1)	−1.6 (−2.1 to −1.1)	<0.001
PBF (%)	43.4 ± 5.4	42.8 ± 5.2	−0.6 (−1.0 to −0.2)	0.009
Fasting glucose (mg/dL)	94 ± 8	89 ± 7	−5 (−9 to −1)	0.030
HbA1c (%)	5.6 ± 0.3	5.5 ± 0.3	−0.1 (NS)	0.421

Data are presented as mean ± SD with 95% CI in parentheses. Paired *t*-tests were used for all variables. BMI: body mass index; PBF: percent body fat; HbA1c: glycated hemoglobin; NS: not significant (*p* > 0.05); CI: confidence interval. HbA1c *p*-value reflects paired *t*-test on available data; minimal change is consistent with near-normal baseline values and diabetes exclusion criteria.

## Data Availability

The datasets generated and analyzed during the current study are available from the corresponding author on reasonable request. Due to privacy regulations and the inclusion of minors, the data are not publicly available.

## Abbreviations

The following abbreviations are used in this manuscript:

Abbreviation	Full Term
ALT	Alanine Aminotransferase
AOMs	Anti-Obesity Medications
AST	Aspartate Aminotransferase
BMI	Body Mass Index
CDC	Centers for Disease Control and Prevention
DXA	Dual-Energy X-ray Absorptiometry
ECLIA	Electrochemiluminescence Immunoassay
ELISA	Enzyme-Linked Immunosorbent Assay
GI	Gastrointestinal
GLP-1	Glucagon-Like Peptide-1
GLP-1 RAs	Glucagon-Like Peptide-1 Receptor Agonists
HbA1c	Glycated Hemoglobin
HDL-C	High-Density Lipoprotein Cholesterol
HOMA-B	Homeostasis Model Assessment of Beta-Cell Function
HOMA-IR	Homeostasis Model Assessment of Insulin Resistance
HPLC	High-Performance Liquid Chromatography
IQR	Interquartile Range
LDL-C	Low-Density Lipoprotein Cholesterol
RCTs	Randomized Controlled Trials
RMR	Resting Metabolic Rate
SDS	Standard Deviation Score
SMM	Skeletal Muscle Mass
TMI	Tri-Ponderal Mass Index
WHO	World Health Organization
